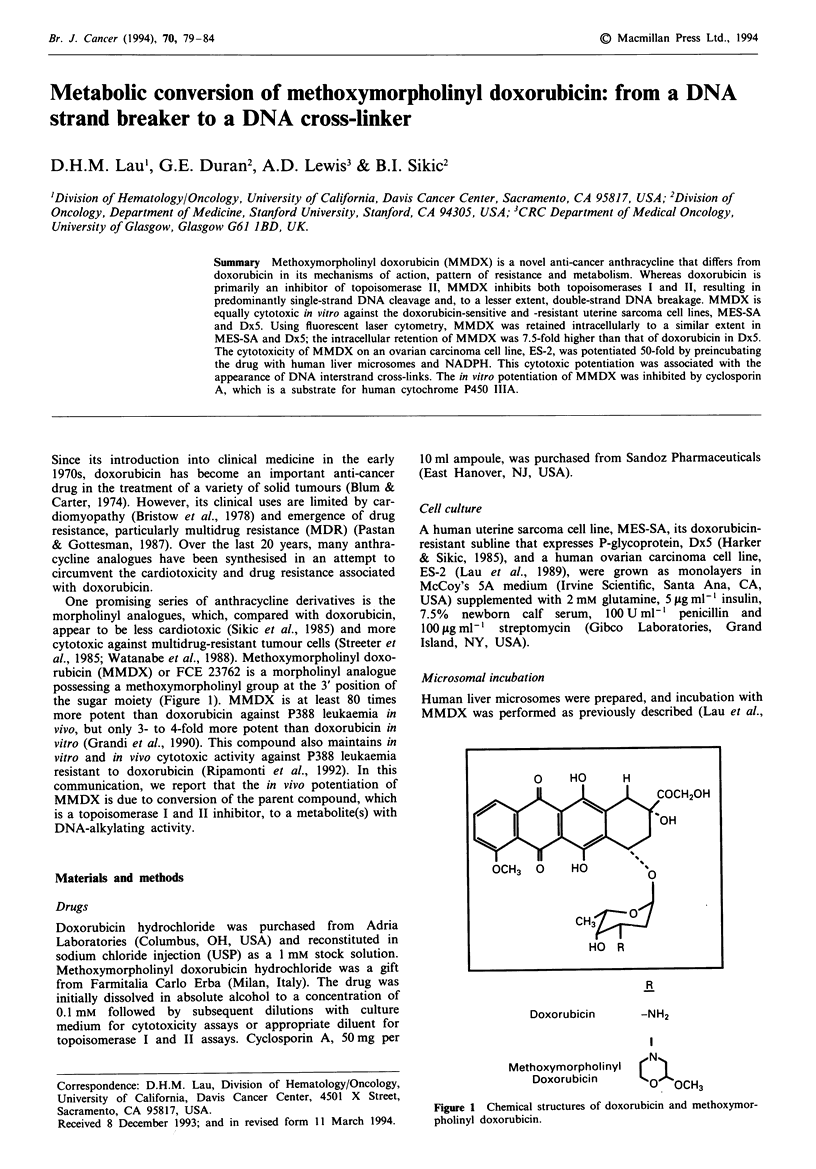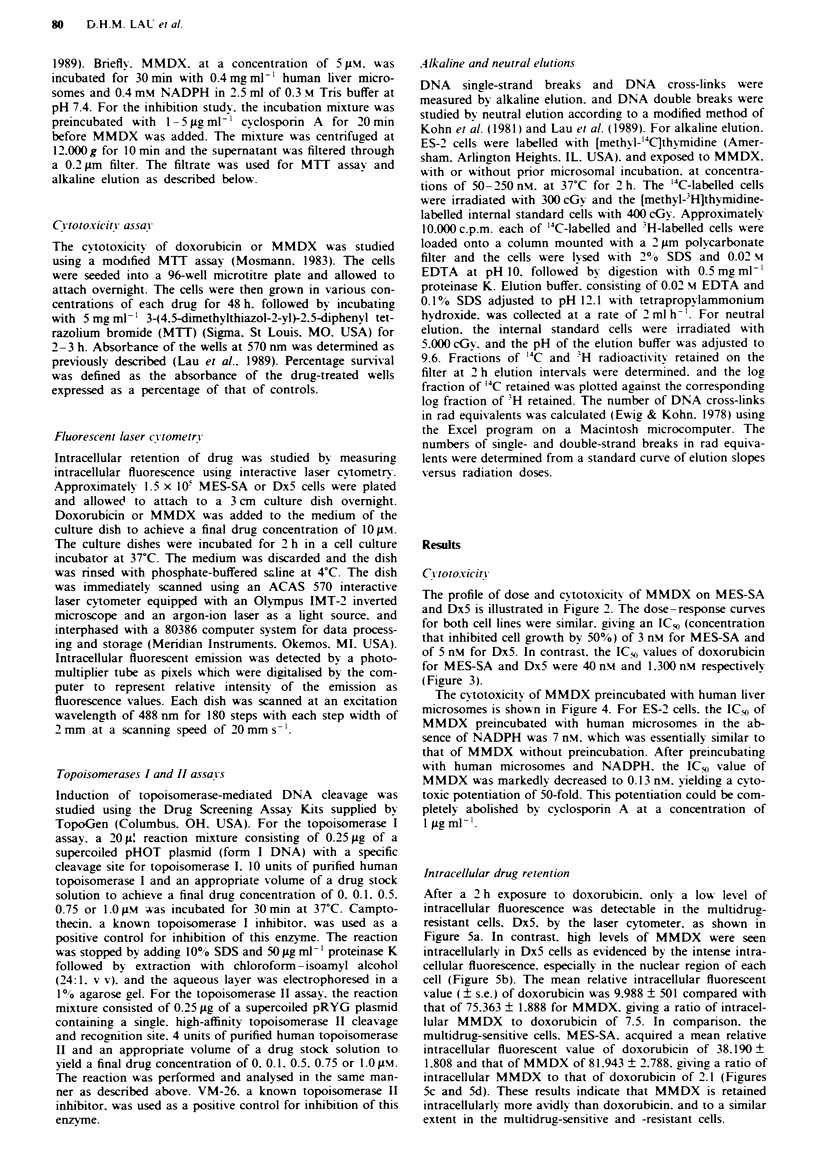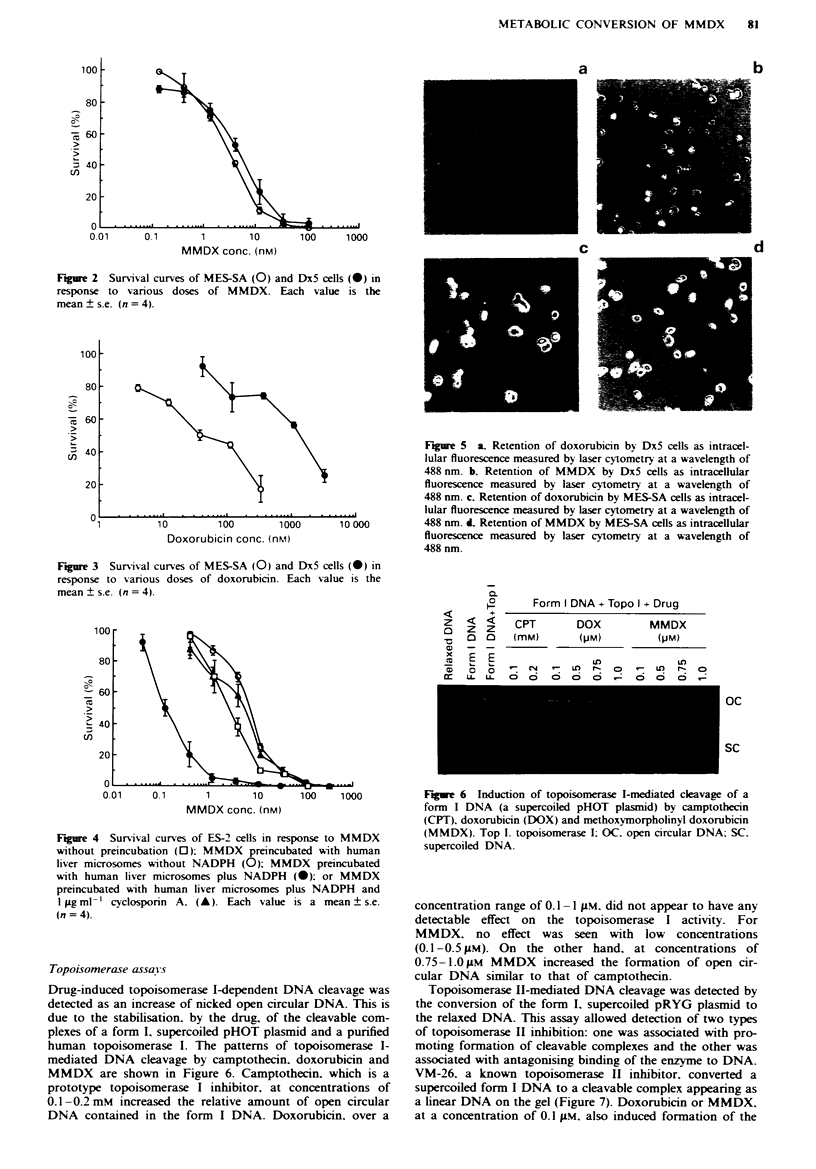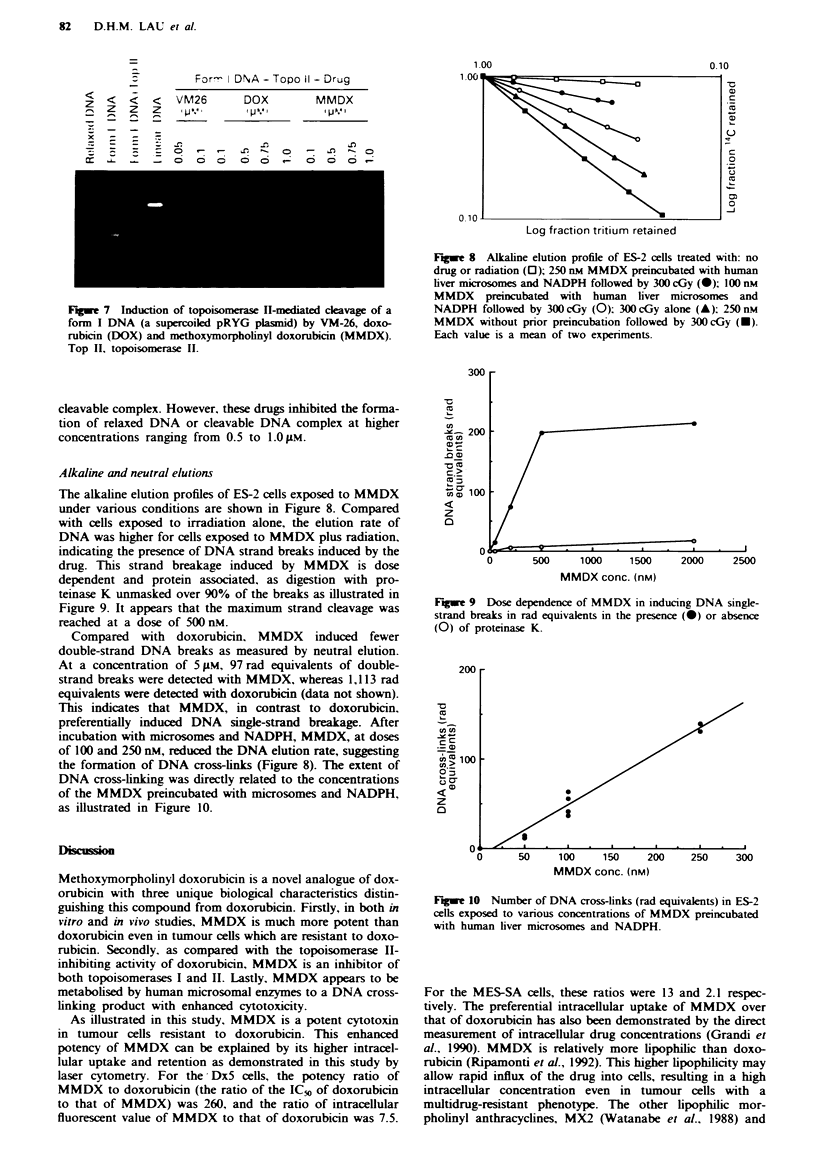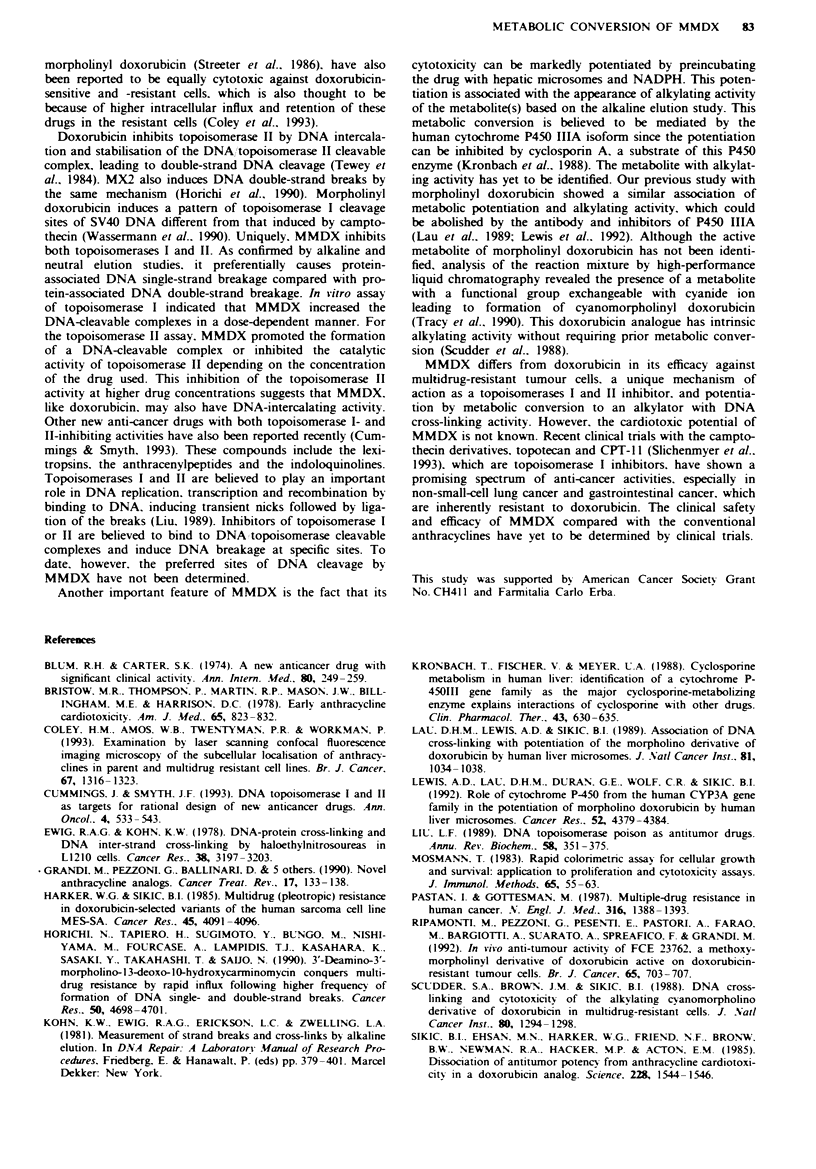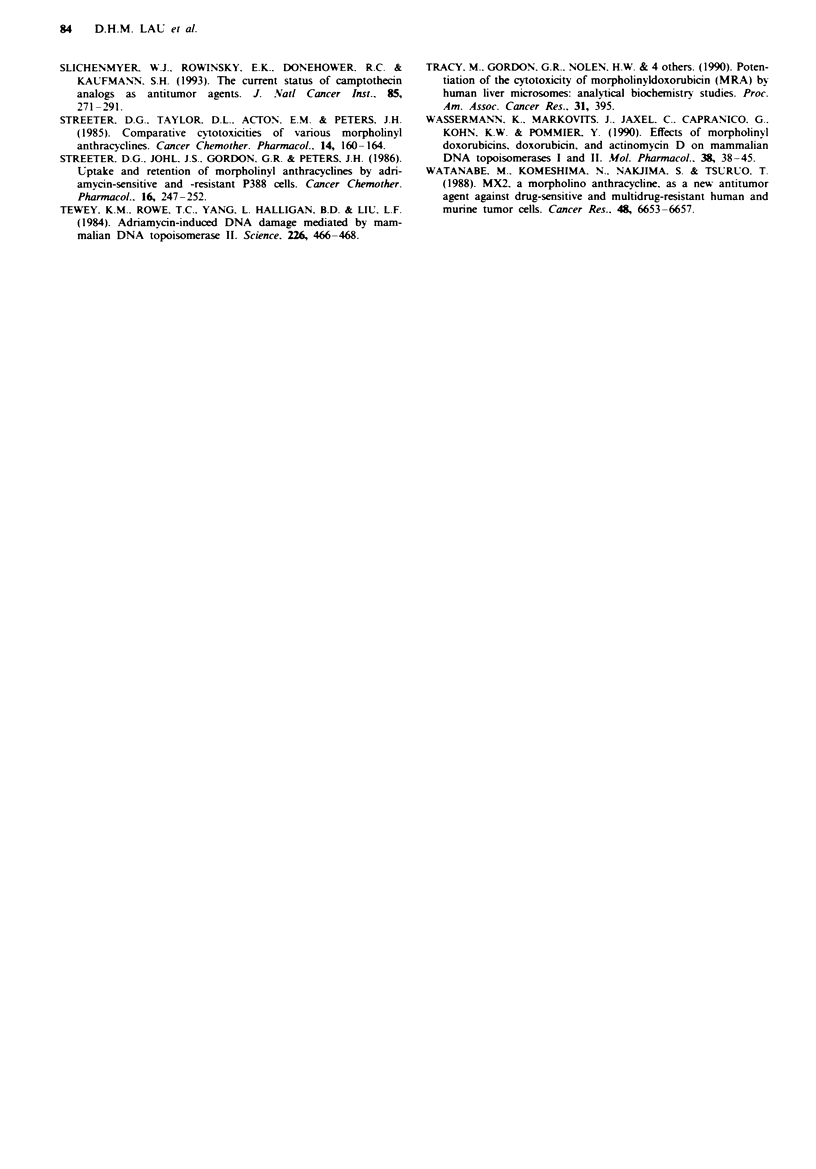# Metabolic conversion of methoxymorpholinyl doxorubicin: from a DNA strand breaker to a DNA cross-linker.

**DOI:** 10.1038/bjc.1994.253

**Published:** 1994-07

**Authors:** D. H. Lau, G. E. Duran, A. D. Lewis, B. I. Sikic

**Affiliations:** Division of Hematology/Oncology, University of California, Davis Cancer Center, Sacramento 95817.

## Abstract

**Images:**


*Br. J. Cancer* (1994), **70**, 79–84 © Macmillan Press Ltd., 1994

### Metabolic conversion of methoxymorpholinyl doxorubicin: from a DNA strand breaker to a DNA cross-linker

D.H.M. Lau[1], G.E. Duran[2], A.D. Lewis[3] & B.I. Sikic[2]

[1]*Division of Hematology/Oncology, University of California, Davis Cancer Center, Sacramento, CA 95817, USA;* [2]*Division of Oncology, Department of Medicine, Stanford University, Stanford, CA 94305, USA;* [3]*CRC Department of Medical Oncology, University of Glasgow, Glasgow G61 1BD, UK.*

**Summary** Methoxymorpholinyl doxorubicin (MMDX) is a novel anti-cancer anthracycline that differs from doxorubicin in its mechanisms of action, pattern of resistance and metabolism. Whereas doxorubicin is primarily an inhibitor of topoisomerase II, MMDX inhibits both topoisomerases I and II, resulting in predominantly single-strand DNA cleavage and, to a lesser extent, double-strand DNA breakage. MMDX is equally cytotoxic *in vitro* against the doxorubicin-sensitive and -resistant uterine sarcoma cell lines, MES-SA and Dx5. Using fluorescent laser cytometry, MMDX was retained intracellularly to a similar extent in MES-SA and Dx5; the intracellular retention of MMDX was 7.5-fold higher than that of doxorubicin in Dx5. The cytotoxicity of MMDX on an ovarian carcinoma cell line, ES-2, was potentiated 50-fold by preincubating the drug with human liver microsomes and NADPH. This cytotoxic potentiation was associated with the appearance of DNA interstrand cross-links. The *in vitro* potentiation of MMDX was inhibited by cyclosporin A, which is a substrate for human cytochrome P450 IIIA.

Since its introduction into clinical medicine in the early 1970s, doxorubicin has become an important anti-cancer drug in the treatment of a variety of solid tumours (Blum & Carter, 1974). However, its clinical uses are limited by cardiomyopathy (Bristow *et al.*, 1978) and emergence of drug resistance, particularly multidrug resistance (MDR) (Pastan & Gottesman, 1987). Over the last 20 years, many anthracycline analogues have been synthesised in an attempt to circumvent the cardiotoxicity and drug resistance associated with doxorubicin.

One promising series of anthracycline derivatives is the morpholinyl analogues, which, compared with doxorubicin, appear to be less cardiotoxic (Sikic *et al.*, 1985) and more cytotoxic against multidrug-resistant tumour cells (Streeter *et al.*, 1985; Watanabe *et al.*, 1988). Methoxymorpholinyl doxorubicin (MMDX) or FCE 23762 is a morpholinyl analogue possessing a methoxymorpholinyl group at the 3′ position of the sugar moiety (Figure 1). MMDX is at least 80 times more potent than doxorubicin against P388 leukaemia *in vivo*, but only 3- to 4-fold more potent than doxorubicin *in vitro* (Grandi *et al.*, 1990). This compound also maintains *in vitro* and *in vivo* cytotoxic activity against P388 leukaemia resistant to doxorubicin (Ripamonti *et al.*, 1992). In this communication, we report that the *in vivo* potentiation of MMDX is due to conversion of the parent compound, which is a topoisomerase I and II inhibitor, to a metabolite(s) with DNA-alkylating activity.

### Materials and methods

### *Drugs*

Doxorubicin hydrochloride was purchased from Adria Laboratories (Columbus, OH, USA) and reconstituted in sodium chloride injection (USP) as a 1 mM stock solution. Methoxymorpholinyl doxorubicin hydrochloride was a gift from Farmitalia Carlo Erba (Milan, Italy). The drug was initially dissolved in absolute alcohol to a concentration of 0.1 mM followed by subsequent dilutions with culture medium for cytotoxicity assays or appropriate diluent for topoisomerase I and II assays. Cyclosporin A, 50 mg per 10 ml ampoule, was purchased from Sandoz Pharmaceuticals (East Hanover, NJ, USA).

### *Cell culture*

A human uterine sarcoma cell line, MES-SA, its doxorubicin-resistant subline that expresses P-glycoprotein, Dx5 (Harker & Sikic, 1985), and a human ovarian carcinoma cell line, ES-2 (Lau *et al.*, 1989), were grown as monolayers in McCoy's 5A medium (Irvine Scientific, Santa Ana, CA, USA) supplemented with 2 mM glutamine, 5 μg $ml^{-1}$ insulin, 7.5% newborn calf serum, 100 U $ml^{-1}$ penicillin and 100 μg $ml^{-1}$ streptomycin (Gibco Laboratories, Grand Island, NY, USA).

### *Microsomal incubation*

Human liver microsomes were prepared, and incubation with MMDX was performed as previously described (Lau *et al.*,

**Figure 1** Chemical structures of doxorubicin and methoxymorpholinyl doxorubicin.

Correspondence: D.H.M. Lau, Division of Hematology/Oncology, University of California, Davis Cancer Center, 4501 X Street, Sacramento, CA 95817, USA.
Received 8 December 1993; and in revised form 11 March 1994.

1989). Briefly, MMDX, at a concentration of 5 μM, was incubated for 30 min with 0.4 mg ml$^{-1}$ human liver microsomes and 0.4 mM NADPH in 2.5 ml of 0.3 M Tris buffer at pH 7.4. For the inhibition study, the incubation mixture was preincubated with 1–5 μg ml$^{-1}$ cyclosporin A for 20 min before MMDX was added. The mixture was centrifuged at 12,000 g for 10 min and the supernatant was filtered through a 0.2 μm filter. The filtrate was used for MTT assay and alkaline elution as described below.

*Cytotoxicity assay*

The cytotoxicity of doxorubicin or MMDX was studied using a modified MTT assay (Mosmann, 1983). The cells were seeded into a 96-well microtitre plate and allowed to attach overnight. The cells were then grown in various concentrations of each drug for 48 h, followed by incubating with 5 mg ml$^{-1}$ 3-(4,5-dimethylthiazol-2-yl)-2,5-diphenyl tetrazolium bromide (MTT) (Sigma, St Louis, MO, USA) for 2–3 h. Absorbance of the wells at 570 nm was determined as previously described (Lau et al., 1989). Percentage survival was defined as the absorbance of the drug-treated wells expressed as a percentage of that of controls.

*Fluorescent laser cytometry*

Intracellular retention of drug was studied by measuring intracellular fluorescence using interactive laser cytometry. Approximately 1.5 × 10$^5$ MES-SA or Dx5 cells were plated and allowed to attach to a 3 cm culture dish overnight. Doxorubicin or MMDX was added to the medium of the culture dish to achieve a final drug concentration of 10 μM. The culture dishes were incubated for 2 h in a cell culture incubator at 37°C. The medium was discarded and the dish was rinsed with phosphate-buffered saline at 4°C. The dish was immediately scanned using an ACAS 570 interactive laser cytometer equipped with an Olympus IMT-2 inverted microscope and an argon-ion laser as a light source, and interphased with a 80386 computer system for data processing and storage (Meridian Instruments, Okemos, MI, USA). Intracellular fluorescent emission was detected by a photomultiplier tube as pixels which were digitalised by the computer to represent relative intensity of the emission as fluorescence values. Each dish was scanned at an excitation wavelength of 488 nm for 180 steps with each step width of 2 mm at a scanning speed of 20 mm s$^{-1}$.

*Topoisomerases I and II assays*

Induction of topoisomerase-mediated DNA cleavage was studied using the Drug Screening Assay Kits supplied by TopoGen (Columbus, OH, USA). For the topoisomerase I assay, a 20 μl reaction mixture consisting of 0.25 μg of a supercoiled pHOT plasmid (form I DNA) with a specific cleavage site for topoisomerase I, 10 units of purified human topoisomerase I and an appropriate volume of a drug stock solution to achieve a final drug concentration of 0, 0.1, 0.5, 0.75 or 1.0 μM was incubated for 30 min at 37°C. Camptothecin, a known topoisomerase I inhibitor, was used as a positive control for inhibition of this enzyme. The reaction was stopped by adding 10% SDS and 50 μg ml$^{-1}$ proteinase K followed by extraction with chloroform–isoamyl alcohol (24:1, v v), and the aqueous layer was electrophoresed in a 1% agarose gel. For the topoisomerase II assay, the reaction mixture consisted of 0.25 μg of a supercoiled pRYG plasmid containing a single, high-affinity topoisomerase II cleavage and recognition site, 4 units of purified human topoisomerase II and an appropriate volume of a drug stock solution to yield a final drug concentration of 0, 0.1, 0.5, 0.75 or 1.0 μM. The reaction was performed and analysed in the same manner as described above. VM-26, a known topoisomerase II inhibitor, was used as a positive control for inhibition of this enzyme.

*Alkaline and neutral elutions*

DNA single-strand breaks and DNA cross-links were measured by alkaline elution, and DNA double breaks were studied by neutral elution according to a modified method of Kohn et al. (1981) and Lau et al. (1989). For alkaline elution, ES-2 cells were labelled with [methyl-$^{14}$C]thymidine (Amersham, Arlington Heights, IL, USA), and exposed to MMDX, with or without prior microsomal incubation, at concentrations of 50–250 nM, at 37°C for 2 h. The $^{14}$C-labelled cells were irradiated with 300 cGy and the [methyl-$^3$H]thymidine-labelled internal standard cells with 400 cGy. Approximately 10,000 c.p.m. each of $^{14}$C-labelled and $^3$H-labelled cells were loaded onto a column mounted with a 2 μm polycarbonate filter and the cells were lysed with 2% SDS and 0.02 M EDTA at pH 10, followed by digestion with 0.5 mg ml$^{-1}$ proteinase K. Elution buffer, consisting of 0.02 M EDTA and 0.1% SDS adjusted to pH 12.1 with tetrapropylammonium hydroxide, was collected at a rate of 2 ml h$^{-1}$. For neutral elution, the internal standard cells were irradiated with 5,000 cGy, and the pH of the elution buffer was adjusted to 9.6. Fractions of $^{14}$C and $^3$H radioactivity retained on the filter at 2 h elution intervals were determined, and the log fraction of $^{14}$C retained was plotted against the corresponding log fraction of $^3$H retained. The number of DNA cross-links in rad equivalents was calculated (Ewig & Kohn, 1978) using the Excel program on a Macintosh microcomputer. The numbers of single- and double-strand breaks in rad equivalents were determined from a standard curve of elution slopes versus radiation doses.

### Results

*Cytotoxicity*

The profile of dose and cytotoxicity of MMDX on MES-SA and Dx5 is illustrated in Figure 2. The dose–response curves for both cell lines were similar, giving an $IC_{50}$ (concentration that inhibited cell growth by 50%) of 3 nM for MES-SA and of 5 nM for Dx5. In contrast, the $IC_{50}$ values of doxorubicin for MES-SA and Dx5 were 40 nM and 1,300 nM respectively (Figure 3).

The cytotoxicity of MMDX preincubated with human liver microsomes is shown in Figure 4. For ES-2 cells, the $IC_{50}$ of MMDX preincubated with human microsomes in the absence of NADPH was 7 nM, which was essentially similar to that of MMDX without preincubation. After preincubating with human microsomes and NADPH, the $IC_{50}$ value of MMDX was markedly decreased to 0.13 nM, yielding a cytotoxic potentiation of 50-fold. This potentiation could be completely abolished by cyclosporin A at a concentration of 1 μg ml$^{-1}$.

*Intracellular drug retention*

After a 2 h exposure to doxorubicin, only a low level of intracellular fluorescence was detectable in the multidrug-resistant cells, Dx5, by the laser cytometer, as shown in Figure 5a. In contrast, high levels of MMDX were seen intracellularly in Dx5 cells as evidenced by the intense intracellular fluorescence, especially in the nuclear region of each cell (Figure 5b). The mean relative intracellular fluorescent value (± s.e.) of doxorubicin was 9,988 ± 501 compared with that of 75,363 ± 1,888 for MMDX, giving a ratio of intracellular MMDX to doxorubicin of 7.5. In comparison, the multidrug-sensitive cells, MES-SA, acquired a mean relative intracellular fluorescent value of doxorubicin of 38,190 ± 1,808 and that of MMDX of 81,943 ± 2,788, giving a ratio of intracellular MMDX to that of doxorubicin of 2.1 (Figures 5c and 5d). These results indicate that MMDX is retained intracellularly more avidly than doxorubicin, and to a similar extent in the multidrug-sensitive and -resistant cells.

**Figure 2** Survival curves of MES-SA (○) and Dx5 cells (●) in response to various doses of MMDX. Each value is the mean ± s.e. ($n = 4$).

**Figure 3** Survival curves of MES-SA (○) and Dx5 cells (●) in response to various doses of doxorubicin. Each value is the mean ± s.e. ($n = 4$).

**Figure 4** Survival curves of ES-2 cells in response to MMDX without preincubation (□); MMDX preincubated with human liver microsomes without NADPH (○); MMDX preincubated with human liver microsomes plus NADPH (●); or MMDX preincubated with human liver microsomes plus NADPH and 1 μg $ml^{-1}$ cyclosporin A. (▲). Each value is a mean ± s.e. ($n = 4$).

**Figure 5** **a**. Retention of doxorubicin by Dx5 cells as intracellular fluorescence measured by laser cytometry at a wavelength of 488 nm. **b**. Retention of MMDX by Dx5 cells as intracellular fluorescence measured by laser cytometry at a wavelength of 488 nm. **c**. Retention of doxorubicin by MES-SA cells as intracellular fluorescence measured by laser cytometry at a wavelength of 488 nm. **d**. Retention of MMDX by MES-SA cells as intracellular fluorescence measured by laser cytometry at a wavelength of 488 nm.

**Figure 6** Induction of topoisomerase I-mediated cleavage of a form I DNA (a supercoiled pHOT plasmid) by camptothecin (CPT), doxorubicin (DOX) and methoxymorpholinyl doxorubicin (MMDX). Top I. topoisomerase I; OC. open circular DNA; SC. supercoiled DNA.

*Topoisomerase assays*

Drug-induced topoisomerase I-dependent DNA cleavage was detected as an increase of nicked open circular DNA. This is due to the stabilisation, by the drug, of the cleavable complexes of a form I, supercoiled pHOT plasmid and a purified human topoisomerase I. The patterns of topoisomerase I-mediated DNA cleavage by camptothecin, doxorubicin and MMDX are shown in Figure 6. Camptothecin, which is a prototype topoisomerase I inhibitor, at concentrations of 0.1–0.2 mM increased the relative amount of open circular DNA contained in the form I DNA. Doxorubicin, over a concentration range of 0.1–1 μM, did not appear to have any detectable effect on the topoisomerase I activity. For MMDX, no effect was seen with low concentrations (0.1–0.5 μM). On the other hand, at concentrations of 0.75–1.0 μM MMDX increased the formation of open circular DNA similar to that of camptothecin.

Topoisomerase II-mediated DNA cleavage was detected by the conversion of the form I, supercoiled pRYG plasmid to the relaxed DNA. This assay allowed detection of two types of topoisomerase II inhibition: one was associated with promoting formation of cleavable complexes and the other was associated with antagonising binding of the enzyme to DNA. VM-26, a known topoisomerase II inhibitor, converted a supercoiled form I DNA to a cleavable complex appearing as a linear DNA on the gel (Figure 7). Doxorubicin or MMDX, at a concentration of 0.1 μM, also induced formation of the

**Figure 7** Induction of topoisomerase II-mediated cleavage of a form I DNA (a supercoiled pRYG plasmid) by VM-26, doxorubicin (DOX) and methoxymorpholinyl doxorubicin (MMDX). Top II, topoisomerase II.

cleavable complex. However, these drugs inhibited the formation of relaxed DNA or cleavable DNA complex at higher concentrations ranging from 0.5 to 1.0 μM.

*Alkaline and neutral elutions*

The alkaline elution profiles of ES-2 cells exposed to MMDX under various conditions are shown in Figure 8. Compared with cells exposed to irradiation alone, the elution rate of DNA was higher for cells exposed to MMDX plus radiation, indicating the presence of DNA strand breaks induced by the drug. This strand breakage induced by MMDX is dose dependent and protein associated, as digestion with proteinase K unmasked over 90% of the breaks as illustrated in Figure 9. It appears that the maximum strand cleavage was reached at a dose of 500 nM.

Compared with doxorubicin, MMDX induced fewer double-strand DNA breaks as measured by neutral elution. At a concentration of 5 μM, 97 rad equivalents of double-strand breaks were detected with MMDX, whereas 1,113 rad equivalents were detected with doxorubicin (data not shown). This indicates that MMDX, in contrast to doxorubicin, preferentially induced DNA single-strand breakage. After incubation with microsomes and NADPH, MMDX, at doses of 100 and 250 nM, reduced the DNA elution rate, suggesting the formation of DNA cross-links (Figure 8). The extent of DNA cross-linking was directly related to the concentrations of the MMDX preincubated with microsomes and NADPH, as illustrated in Figure 10.

### Discussion

Methoxymorpholinyl doxorubicin is a novel analogue of doxorubicin with three unique biological characteristics distinguishing this compound from doxorubicin. Firstly, in both *in vitro* and *in vivo* studies, MMDX is much more potent than doxorubicin even in tumour cells which are resistant to doxorubicin. Secondly, as compared with the topoisomerase II-inhibiting activity of doxorubicin, MMDX is an inhibitor of both topoisomerases I and II. Lastly, MMDX appears to be metabolised by human microsomal enzymes to a DNA cross-linking product with enhanced cytotoxicity.

As illustrated in this study, MMDX is a potent cytotoxin in tumour cells resistant to doxorubicin. This enhanced potency of MMDX can be explained by its higher intracellular uptake and retention as demonstrated in this study by laser cytometry. For the Dx5 cells, the potency ratio of MMDX to doxorubicin (the ratio of the $IC_{50}$ of doxorubicin to that of MMDX) was 260, and the ratio of intracellular fluorescent value of MMDX to that of doxorubicin was 7.5.

**Figure 8** Alkaline elution profile of ES-2 cells treated with: no drug or radiation (□); 250 nM MMDX preincubated with human liver microsomes and NADPH followed by 300 cGy (●); 100 nM MMDX preincubated with human liver microsomes and NADPH followed by 300 cGy (○); 300 cGy alone (▲); 250 nM MMDX without prior preincubation followed by 300 cGy (■). Each value is a mean of two experiments.

**Figure 9** Dose dependence of MMDX in inducing DNA single-strand breaks in rad equivalents in the presence (●) or absence (○) of proteinase K.

**Figure 10** Number of DNA cross-links (rad equivalents) in ES-2 cells exposed to various concentrations of MMDX preincubated with human liver microsomes and NADPH.

For the MES-SA cells, these ratios were 13 and 2.1 respectively. The preferential intracellular uptake of MMDX over that of doxorubicin has also been demonstrated by the direct measurement of intracellular drug concentrations (Grandi *et al.*, 1990). MMDX is relatively more lipophilic than doxorubicin (Ripamonti *et al.*, 1992). This higher lipophilicity may allow rapid influx of the drug into cells, resulting in a high intracellular concentration even in tumour cells with a multidrug-resistant phenotype. The other lipophilic morpholinyl anthracyclines, MX2 (Watanabe *et al.*, 1988) and morpholinyl doxorubicin (Streeter *et al.*, 1986), have also been reported to be equally cytotoxic against doxorubicin-sensitive and -resistant cells, which is also thought to be because of higher intracellular influx and retention of these drugs in the resistant cells (Coley *et al.*, 1993).

Doxorubicin inhibits topoisomerase II by DNA intercalation and stabilisation of the DNA/topoisomerase II cleavable complex, leading to double-strand DNA cleavage (Tewey *et al.*, 1984). MX2 also induces DNA double-strand breaks by the same mechanism (Horichi *et al.*, 1990). Morpholinyl doxorubicin induces a pattern of topoisomerase I cleavage sites of SV40 DNA different from that induced by camptothecin (Wassermann *et al.*, 1990). Uniquely, MMDX inhibits both topoisomerases I and II. As confirmed by alkaline and neutral elution studies, it preferentially causes protein-associated DNA single-strand breakage compared with protein-associated DNA double-strand breakage. *In vitro* assay of topoisomerase I indicated that MMDX increased the DNA-cleavable complexes in a dose-dependent manner. For the topoisomerase II assay, MMDX promoted the formation of a DNA-cleavable complex or inhibited the catalytic activity of topoisomerase II depending on the concentration of the drug used. This inhibition of the topoisomerase II activity at higher drug concentrations suggests that MMDX, like doxorubicin, may also have DNA-intercalating activity. Other new anti-cancer drugs with both topoisomerase I- and II-inhibiting activities have also been reported recently (Cummings & Smyth, 1993). These compounds include the lexitropsins, the anthracenylpeptides and the indoloquinolines. Topoisomerases I and II are believed to play an important role in DNA replication, transcription and recombination by binding to DNA, inducing transient nicks followed by ligation of the breaks (Liu, 1989). Inhibitors of topoisomerase I or II are believed to bind to DNA/topoisomerase cleavable complexes and induce DNA breakage at specific sites. To date, however, the preferred sites of DNA cleavage by MMDX have not been determined.

Another important feature of MMDX is the fact that its cytotoxicity can be markedly potentiated by preincubating the drug with hepatic microsomes and NADPH. This potentiation is associated with the appearance of alkylating activity of the metabolite(s) based on the alkaline elution study. This metabolic conversion is believed to be mediated by the human cytochrome P450 IIIA isoform since the potentiation can be inhibited by cyclosporin A, a substrate of this P450 enzyme (Kronbach *et al.*, 1988). The metabolite with alkylating activity has yet to be identified. Our previous study with morpholinyl doxorubicin showed a similar association of metabolic potentiation and alkylating activity, which could be abolished by the antibody and inhibitors of P450 IIIA (Lau *et al.*, 1989; Lewis *et al.*, 1992). Although the active metabolite of morpholinyl doxorubicin has not been identified, analysis of the reaction mixture by high-performance liquid chromatography revealed the presence of a metabolite with a functional group exchangeable with cyanide ion leading to formation of cyanomorpholinyl doxorubicin (Tracy *et al.*, 1990). This doxorubicin analogue has intrinsic alkylating activity without requiring prior metabolic conversion (Scudder *et al.*, 1988).

MMDX differs from doxorubicin in its efficacy against multidrug-resistant tumour cells, a unique mechanism of action as a topoisomerases I and II inhibitor, and potentiation by metabolic conversion to an alkylator with DNA cross-linking activity. However, the cardiotoxic potential of MMDX is not known. Recent clinical trials with the camptothecin derivatives, topotecan and CPT-11 (Slichenmyer *et al.*, 1993), which are topoisomerase I inhibitors, have shown a promising spectrum of anti-cancer activities, especially in non-small-cell lung cancer and gastrointestinal cancer, which are inherently resistant to doxorubicin. The clinical safety and efficacy of MMDX compared with the conventional anthracyclines have yet to be determined by clinical trials.

This study was supported by American Cancer Society Grant No. CH411 and Farmitalia Carlo Erba.

### References

BLUM, R.H. & CARTER, S.K. (1974). A new anticancer drug with significant clinical activity. *Ann. Intern. Med.*, **80**, 249-259.

BRISTOW, M.R., THOMPSON, P., MARTIN, R.P., MASON, J.W., BILLINGHAM, M.E. & HARRISON, D.C. (1978). Early anthracycline cardiotoxicity. *Am. J. Med.*, **65**, 823-832.

COLEY, H.M., AMOS, W.B., TWENTYMAN, P.R. & WORKMAN, P. (1993). Examination by laser scanning confocal fluorescence imaging microscopy of the subcellular localisation of anthracyclines in parent and multidrug resistant cell lines. *Br. J. Cancer*, **67**, 1316-1323.

CUMMINGS, J. & SMYTH, J.F. (1993). DNA topoisomerase I and II as targets for rational design of new anticancer drugs. *Ann. Oncol.*, **4**, 533-543.

EWIG, R.A.G. & KOHN, K.W. (1978). DNA-protein cross-linking and DNA inter-strand cross-linking by haloethylnitrosoureas in L1210 cells. *Cancer Res.*, **38**, 3197-3203.

GRANDI, M., PEZZONI, G., BALLINARI, D. & 5 others. (1990). Novel anthracycline analogs. *Cancer Treat. Rev.*, **17**, 133-138.

HARKER, W.G. & SIKIC, B.I. (1985). Multidrug (pleotropic) resistance in doxorubicin-selected variants of the human sarcoma cell line MES-SA. *Cancer Res.*, **45**, 4091-4096.

HORICHI, N., TAPIERO, H., SUGIMOTO, Y., BUNGO, M., NISHIYAMA, M., FOURCASE, A., LAMPIDIS, T.J., KASAHARA, K., SASAKI, Y., TAKAHASHI, T. & SAIJO, N. (1990). 3'-Deamino-3'-morpholino-13-deoxo-10-hydroxycarminomycin conquers multidrug resistance by rapid influx following higher frequency of formation of DNA single- and double-strand breaks. *Cancer Res.*, **50**, 4698-4701.

KOHN, K.W., EWIG, R.A.G., ERICKSON, L.C. & ZWELLING, L.A. (1981). Measurement of strand breaks and cross-links by alkaline elution. In *DNA Repair: A Laboratory Manual of Research Procedures*, Friedberg, E. & Hanawalt, P. (eds) pp. 379-401. Marcel Dekker: New York.

KRONBACH, T., FISCHER, V. & MEYER, U.A. (1988). Cyclosporine metabolism in human liver: identification of a cytochrome P-450III gene family as the major cyclosporine-metabolizing enzyme explains interactions of cyclosporine with other drugs. *Clin. Pharmacol. Ther.*, **43**, 630-635.

LAU, D.H.M., LEWIS, A.D. & SIKIC, B.I. (1989). Association of DNA cross-linking with potentiation of the morpholino derivative of doxorubicin by human liver microsomes. *J. Natl Cancer Inst.*, **81**, 1034-1038.

LEWIS, A.D., LAU, D.H.M., DURAN, G.E., WOLF, C.R. & SIKIC, B.I. (1992). Role of cytochrome P-450 from the human CYP3A gene family in the potentiation of morpholino doxorubicin by human liver microsomes. *Cancer Res.*, **52**, 4379-4384.

LIU, L.F. (1989). DNA topoisomerase poison as antitumor drugs. *Annu. Rev. Biochem.*, **58**, 351-375.

MOSMANN, T. (1983). Rapid colorimetric assay for cellular growth and survival: application to proliferation and cytotoxicity assays. *J. Immunol. Methods*, **65**, 55-63.

PASTAN, I. & GOTTESMAN, M. (1987). Multiple-drug resistance in human cancer. *N. Engl. J. Med.*, **316**, 1388-1393.

RIPAMONTI, M., PEZZONI, G., PESENTI, E., PASTORI, A., FARAO, M., BARGIOTTI, A., SUARATO, A., SPREAFICO, F. & GRANDI, M. (1992). *In vivo* anti-tumour activity of FCE 23762, a methoxymorpholinyl derivative of doxorubicin active on doxorubicin-resistant tumour cells. *Br. J. Cancer*, **65**, 703-707.

SCUDDER, S.A., BROWN, J.M. & SIKIC, B.I. (1988). DNA cross-linking and cytotoxicity of the alkylating cyanomorpholino derivative of doxorubicin in multidrug-resistant cells. *J. Natl Cancer Inst.*, **80**, 1294-1298.

SIKIC, B.I., EHSAN, M.N., HARKER, W.G., FRIEND, N.F., BRONW, B.W., NEWMAN, R.A., HACKER, M.P. & ACTON, E.M. (1985). Dissociation of antitumor potency from anthracycline cardiotoxicity in a doxorubicin analog. *Science*, **228**, 1544-1546.

SLICHENMYER, W.J., ROWINSKY, E.K., DONEHOWER, R.C. & KAUFMANN, S.H. (1993). The current status of camptothecin analogs as antitumor agents. *J. Natl Cancer Inst.*, **85**, 271-291.

STREETER, D.G., TAYLOR, D.L., ACTON, E.M. & PETERS, J.H. (1985). Comparative cytotoxicities of various morpholinyl anthracyclines. *Cancer Chemother. Pharmacol.*, **14**, 160-164.

STREETER, D.G., JOHL, J.S., GORDON, G.R. & PETERS, J.H. (1986). Uptake and retention of morpholinyl anthracyclines by adriamycin-sensitive and -resistant P388 cells. *Cancer Chemother. Pharmacol.*, **16**, 247-252.

TEWEY, K.M., ROWE, T.C., YANG, L. HALLIGAN, B.D. & LIU, L.F. (1984). Adriamycin-induced DNA damage mediated by mammalian DNA topoisomerase II. *Science*, **226**, 466-468.

TRACY, M., GORDON, G.R., NOLEN, H.W. & 4 others. (1990). Potentiation of the cytotoxicity of morpholinyldoxorubicin (MRA) by human liver microsomes: analytical biochemistry studies. *Proc. Am. Assoc. Cancer Res.*, **31**, 395.

WASSERMANN, K., MARKOVITS, J., JAXEL, C., CAPRANICO, G., KOHN, K.W. & POMMIER, Y. (1990). Effects of morpholinyl doxorubicins, doxorubicin, and actinomycin D on mammalian DNA topoisomerases I and II. *Mol. Pharmacol.*, **38**, 38-45.

WATANABE, M., KOMESHIMA, N., NAKJIMA, S. & TSURUO, T. (1988). MX2, a morpholino anthracycline, as a new antitumor agent against drug-sensitive and multidrug-resistant human and murine tumor cells. *Cancer Res.*, **48**, 6653-6657.